# 520. Development of Cefepime-taniborbactam MIC Antimicrobial Susceptibility Test (AST) for Enterobacterales and *Pseudomonas aeruginosa* on MicroScan Dried Gram-negative MIC Panels

**DOI:** 10.1093/ofid/ofac492.575

**Published:** 2022-12-15

**Authors:** Enrique J Fernandez, Robert L Williams, Vasna Carr, Vasna Carr, Miller Renae

**Affiliations:** Beckman Coulter, Inc., West Sacramento, California; Beckman Coulter, Inc., West Sacramento, California; Beckman Coulter, Inc., West Sacramento, California; Beckman Coulter, Inc., West Sacramento, California; Beckman Coulter, Inc., West Sacramento, California

## Abstract

**Background:**

Automated AST devices are critical to inform appropriate care of patients with bacterial infections. Cefepime-taniborbactam is an investigational agent under development to treat infections due to multidrug-resistant bacteria, including carbapenem-resistant Enterobacterales and *Pseudomonas aeruginosa*. Development of a cefepime-taniborbactam antimicrobial susceptibility test was completed for the MicroScan Dried Gram-negative MIC (MSDGN) Panel when compared to CLSI broth microdilution reference panels.

**Methods:**

Development was conducted by comparing MICs obtained using the MSDGN panel to MICs using a CLSI broth microdilution reference panel. The concentration range of cefepime-taniborbactam evaluated was 0.12/4-64/4 µg/mL. A total of 1376 isolates (1256 Enterobacterales and 120 *P. aeruginosa*) were tested at 16, 18, and 20 hours incubation times (for visual read and autoSCAN-4). A total of 917 isolates (837 Enterobacterales and 80 *P. aeruginosa*) were tested at 16 and 18 hours incubation time for the WalkAway System. All isolates were tested using turbidity and Prompt^®^ methods of inoculation. MSDGN panels were incubated at 35 ± 1^°^C. Reference panels, prepared according to ISO methodology, were inoculated using the turbidity inoculation method. All frozen reference panels were incubated at 35 ± 2°C and read visually. Rates of essential agreement (MicroScan MIC within ± 1 doubling dilution of reference MIC) were determined for each combination of inoculation method and read method.

**Results:**

Essential agreement rates ranged from 93.0% to 98.8% for all isolates tested during development. Essential agreement rates for each inoculation and read method are presented in Table 1.

**Table 1:**
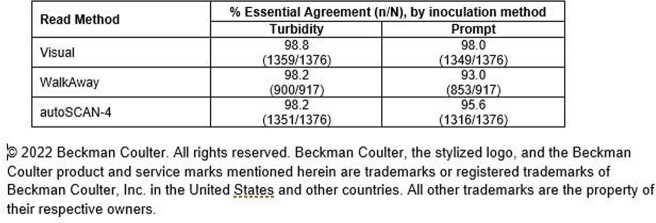
- Results

**Conclusion:**

Cefepime-taniborbactam MIC results obtained with the MSDGN panel correlated well with MIC results obtained using reference panels. Rates of essential agreement were ≥93.0% for all inoculation and read methods. These development data support the continued evaluation of MSDGN panel with cefepime-taniborbactam in a multicenter trial.

Pending submission and clearance by the United States Food and Drug Administration; not yet available for in vitro diagnostic use in the US. For Investigational Use Only. The performance characteristics of this product have not been established.

**Disclosures:**

**Enrique J. Fernandez, BS**, Beckman Coulter, Inc.: Employee of Beckman Coulter **Robert L. Williams, PhD**, Beckman Coulter, Inc.: Employee of Beckman Coulter **Vasna Carr, BS**, Beckman Coulter, Inc.: Employee of Beckman Coulter **Vasna Carr, BS**, Beckman Coulter, Inc.: Employee of Beckman Coulter **Miller Renae, BS**, Beckman Coulter, Inc.: Employee of Beckman Coulter.

